# Reliability Analysis of PAUT Based on the Round-Robin Test for Pipe Welds with Thermal Fatigue Cracks

**DOI:** 10.3390/ma16216908

**Published:** 2023-10-27

**Authors:** Dongchan Kang, Yu Min Choi, Dong Min Lee, Jung Bin Kim, Yong Kwon Kim, Tae Sung Park, Ik Keun Park

**Affiliations:** 1 NDT Research Center, Seoul National University of Science and Technology, 232 Gongneung-ro, Nowon-gu, Seoul 01811, Gyeonggi-do, Republic of Korea; dongchan@seoultech.ac.kr; 2 Doosan Enerbility, 22, Doosan Volvo-ro, Seongsan-gu, Changwon-si 51711, Gyeongsangnam-do, Republic of Korea; yumin.choi@doosan.com; 3 KEPCO KPS, 211 Munhwa-ro, Naju-si 58326, Jeolla-do, Republic of Korea; dmlee@kps.co.kr (D.M.L.); jbkim@kps.co.kr (J.B.K.); ykkim76@kps.co.kr (Y.K.K.); tpark@kps.co.kr (T.S.P.)

**Keywords:** thermal fatigue crack, phased array ultrasonic testing, probability of detection

## Abstract

Thermal fatigue cracks occurring in pipes in nuclear power plants pose a high degree of risk. Thermal fatigue cracks are generated when the thermal fatigue load caused by local temperature gradients is repeatedly applied. The flaws are mainly found in welds, owing to the effects of stress concentration caused by the material properties and geometric shapes of welds. Thermal fatigue pipes are classified as targets of risk-informed in-service inspection, for which ultrasonic testing, a volumetric non-destructive testing method, is applied. With the advancement of ultrasonic testing techniques, various studies have been conducted recently to apply the phased array ultrasonic testing (PAUT) method to the inspection of thermal fatigue cracks occurring on pipes. A quantitative reliability analysis of the PAUT method must be performed to apply the PAUT method to on-site thermal fatigue crack inspection. In this study, to evaluate the quantitative reliability of the PAUT method for thermal fatigue cracks, we fabricated crack specimens with the thermal fatigue mechanism applied to the pipe welds. We performed a round-robin test to collect PAUT data and determine the validity of the detection performance (probability of detection; POD) and the error in the sizing accuracy (root-mean-square error; RMSE) evaluation. The analysis results of the POD and sizing performance of the length and depth of thermal fatigue cracks were comparatively evaluated with the acceptance criteria of the American Society of Mechanical Engineers Code to confirm the effectiveness of applying the PAUT method.

## 1. Introduction

Thermal fatigue cracks occur when the thermal fatigue load caused by the local temperature gradient is repeated. Thermal stratification is a phenomenon caused by density differences in a layer of hot fluid flowing over a layer of cold fluid. This thermal stratification is a problem in many piping systems used in NPP where hot and cold fluids coexist or are separated by leakage valves. The main equipment of a nuclear power plant include a pressurizer surge line, a reactor coolant system branch line, a shutdown cooling system, and a mixing tee; within the equipment, low-temperature and high-temperature fluids are mixed or coexist. Hence, such equipment operate in conditions where thermal fatigue cracks can easily form [1,2,3]. Studies related to thermal stratification due to sources of thermal fatigue crack generation and fatigue life evaluation using simulations have been conducted on thermal and mechanical analyses of the effects of non-uniform temperature regions inside pipes due to thermal stratification [4,5,6]. Thermal fatigue cracks in nuclear power plants are damages that are not considered in the design stage. Owing to an increase in the number of thermally fatigued pipes, they have been classified as a target of risk-informed in-service inspection (RI-ISI). Moreover, an inspection cycle based on the cumulative fatigue coefficient has been applied [3]. A non-destructive volumetric test is applied to thermal fatigue pipes according to the American Society of Mechanical Engineers (ASME) Boiler and Pressure Vessel Code [7].

Typical non-destructive volumetric testing methods include radiographic testing (RT) and ultrasonic testing (UT). RT has an excellent recording characteristic and high accuracy in identifying volumetric flaws, such as porosity and slag inclusions. However, when applying RT, there is a risk that inspectors and power plant employees may be exposed to radiation since the inspection is carried out in a location where the radiation source is exposed. Furthermore, its application is limited during ISI because of the restriction of access to the work area and the limitation of radiation intensity [8]. Recently, with the rapid advancement of ultrasonic testing (UT) techniques, various studies have been conducted on UT to replace RT. The Electric Power Research Institute (EPRI) has been developing various code cases by designing and fabricating pipe weld specimens and performing UT as technical grounds for replacing volumetric inspections from RT to UT [9,10]. Furthermore, UT is applied in ISI because it is advantageous for the detection of cracks, which are difficult to detect with RT.

However, conventional UT can be subject to errors in signal analysis and determination results because of basic limitations, such as the diffraction, attenuation, refraction, reflection, dead zone, waveform variation, spurious echo, and transmission loss of ultrasound waves. Furthermore, UT has lower reliability and recordability than RT, a lack of equipment and specimen reliability, and insufficient development of inspection technology and standards. In addition, there are limitations depending on the geometry of the specimen such as taper and curvature in the pipe weld, and it is difficult for an inspector to recognize flaws and accurately determine the flaw type if there is no information on the design parameters or actual dimensions of the geometrics of the pipe [11]. Additionally, comparative evaluations using EDM notches are being conducted to evaluate these phenomena.

In recent years, studies have been conducted to apply various testing methods for a more accurate evaluation of thermal fatigue cracks. These include studies on the time-of-flight diffraction (TOFD) technique, which evaluates flaws using the diffraction signals of ultrasounds [12,13], and the evaluation of the flaw depth using eddy current inspection (ECT) [14,15]. Testing methods using TOFD or ECT have difficulties in analyzing signals according to the geometry of the welds and quantitatively evaluating the flaw height. In contrast, the PAUT method is excellent in terms of crack detection and can evaluate the flaw size (length and height) with high accuracy. Thus, research and empirical verification are underway to apply this method to the ISI of major components in nuclear power plants [16]. However, most studies remain at the level of verifying the performance of inspection equipment or inspection methods. To apply the PAUT method to thermal fatigue cracks, a quantitative reliability evaluation must be performed, such as that for the probability of detection (POD) and size measurement accuracy for the overall inspection system, including human factors.

In this study, we evaluate the quantitative reliability of the PAUT method for thermal fatigue cracks. We fabricated thermal fatigue crack specimens, which simulated a nuclear plant-like environment of welded parts, and performed a round-robin test (RRT) to consider human factors. Using the PAUT data acquired through the RRT, we calculated the POD and sizing performance using statistical analyses. The reliability analysis results were comparatively evaluated using the acceptance criteria of the AMSE Code to establish a technical basis for the application of the PAUT method to pipe welds with thermal fatigue cracks.

## 2. Experimental Set-Up and Data Acquisition

### 2.1. Fabrication of Thermal Fatigue Crack Specimens

**Figure 1:**
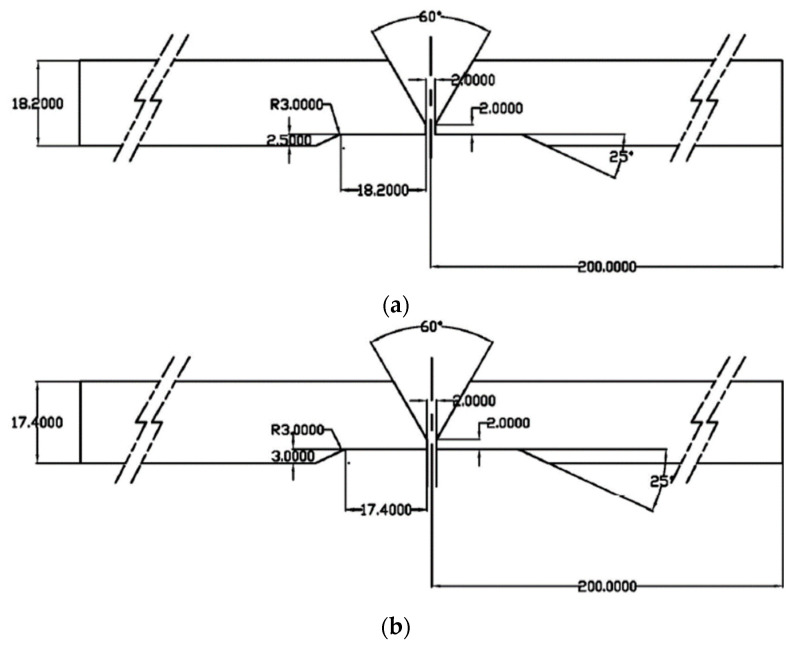
Blueprints of the thermal fatigue pipe weld specimens [mm]: (**a**) outer diameter 203.2 mm, thickness 18.2 mm; and (**b**) outer diameter 304.8 mm, thickness 17.4 mm.

To evaluate the reliability of the PAUT method for thermal fatigue pipes, we fabricated flaw specimens in which cracks were grown under thermal fatigue load as in the nuclear power plant environment. The specimens were carbon steel butt welds of pipes of two diameters, 203.2 mm and 304.8 mm, with a thickness of 18.2 mm and 17.4 mm, respectively. We chose butt welds for the base material of the pipe welds, processed with counter bores. Thermal fatigue cracks can easily occur on a counterbore because of high stress and the geometric characteristics of the pipe [17]. Figure 1 shows blueprints of the fabricated specimens for the thermal fatigue pipe flaw investigation.

**Figure 2:**
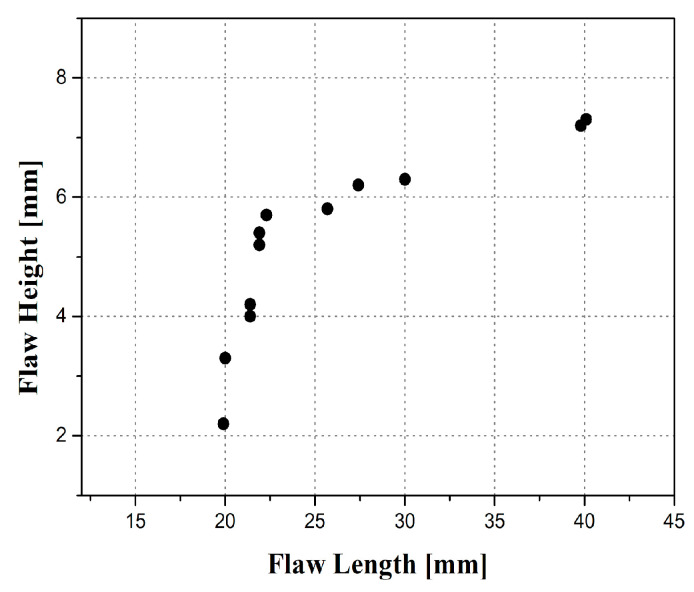
Length and depth distribution of thermal fatigue cracks.

Thermal fatigue cracks were fabricated by applying tensile stress using a jig, raising the temperature to a maximum of 400 °C, holding it for approximately one minute, water-cooling to about 20 °C, and repeating it 100 times or more to grow thermal fatigue cracks. A total of 12 flaws were divided and inserted into five specimens to avoid interference between the flaws. The flaws were designed to be positioned within the weld volume corresponding to the inspected volume: a range of 1/4 in from the adjacent base material and 1/3 of the thickness at the bottom. Each flaw was designed with a different length and depth, which was an analysis condition for quantitative reliability evaluation. The length and depth of the flaw were designed to be evenly distributed between 20 mm and 40 mm and between 2 mm and 7.3 mm, respectively. Figure 2 shows the length and depth distribution of the flaws.

### 2.2. PAUT Inspection System and Procedure

We used the ZETEC TOPAZ64 (Snoqualmie, WA, USA) and the Olympus X3(MX2) (Tokyo, Japan) as the inspection equipment for data acquisition in the RRT, which provide more than 32 channels. We used a probe with a 5 MHz frequency, 32 oscillators, and 0.6 mm pitch, which is widely used for pipe inspection. The wedge had a shear-wave refraction angle of 55°, with a curvature processed in such a way that the space between the inspected surfaces did not exceed 0.5 mm. An encoder-mounted automatic/semi-automatic scanner was used to acquire data. The essential variables of the scan plan for the sectorial scan were the reference point of the weld axis, weld geometrics, and sweep angle; the index offset must be set up to include all inspection volumes. Table 1 below summarizes the specifics.

**Figure 3:**
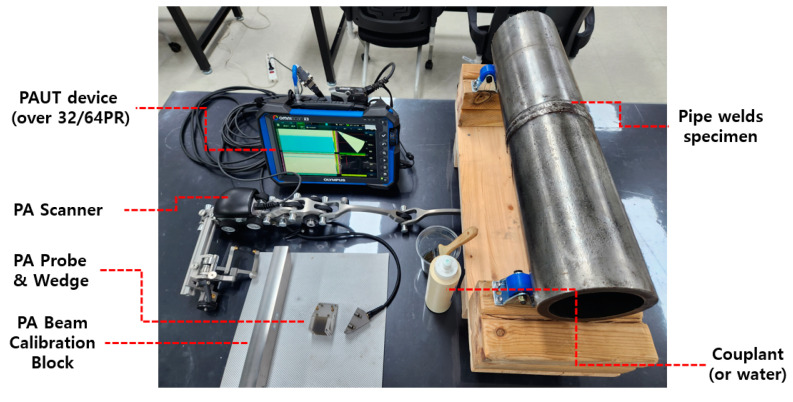
Schematic diagram of PAUT system for RRT.

Calibration of the ultrasonic beam and time-corrected gain (TCG) was performed on the equipment used for the inspection. We set the reference sensitivity to 100% of the full-screen height (FSH) for the notch of the calibration specimen, which has the same diameter and thickness as the specimens. Using the evaluation criteria of the PAUT, we performed this investigation for all indications exceeding 10% of the reference sensitivity set up previously and outlined and determined the presence or absence of flaws through signal analysis. The tip diffraction method was applied as the main measurement method to measure the height of the flaw, but when the tip signals could not be distinguished, the 3 dB drop method was applied as an alternative method. The length of the flaw was measured using the 6 dB drop method. A schematic diagram of the entire system used in the RRT is shown in Figure 3.

### 2.3. Round-Robin Test for Data Acquisition

To validate the prepared inspection system and procedure, we performed a round-robin test (RRT), which is typically used in reliability evaluations of non-destructive testing [18]. We performed a blind RRT, in which flaw information is not provided, to evaluate the human factors of the inspectors, as well as the PAUT method. The inspectors who participated in the test had a qualification of UT/PAUT level II or higher.

In this study, the RRT was performed on three teams that met the above qualifications and each included at least one inspector with more than 10 years of experience. The RRT was performed as a blind RRT that did not disclose defect information of the test specimen in order to simultaneously evaluate the influence of the inspector’s skill level. All participating inspection teams acquired PAUT data according to the inspection equipment and procedures presented in Section 2.2. The acquired data were written in a report in the same format, and detailed information such as the number, location, length, and depth of defects was collected for reliability analysis.

**Figure 4:**
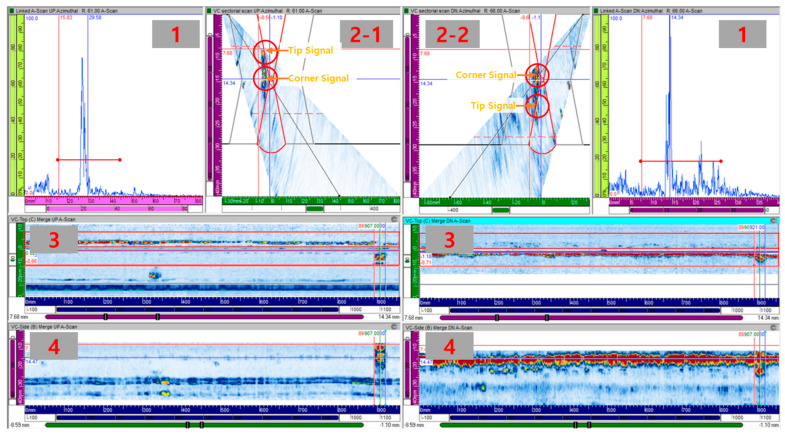
Example of the PAUT data of thermal fatigue crack by RRT.

An example of data acquired with the RRT is shown in Figure 4. The windows arranged symmetrically on the left and right indicate data collected from the left or right side of the weld area. The data shown in each window are as follows: No. 1. A-scan (pulse-echo) signal at the selected angle, No. 2. sectorial scan image, No. 3. C-scan image, and No. 4. B-scan image. The length of the defect in the data is measured by moving the data in the inspection direction from the point where the A-scan signal is at the maximum and measuring the distance between the points where the maximum signal is half (i.e., −6 dB). The tip diffraction technique is applied to measure the height of a defect by measuring the distance between the edge signal of the defect and the diffraction signal appearing at the tip of the defect. Examples of corner and tip signals are shown in the S-scan image in Figure 4 (No. 2-1, 2-2). If no tip signal is present, the distance between half (−6 dB) or 0.7 times (−3 dB) the maximum signal is measured, similar to measuring length.

## 3. Reliability Analysis of PAUT Data

Indicators for evaluating the reliability of the non-destructive test include the POD and sizing performance (root mean square; RMS). The POD is classified into the binary POD model, which is based on hit/miss data, and the a^ vs. a POD model, which is based on the signal response data (a^) of the flaw size (a) [19]. Each model chooses and applies an appropriate method according to the inspection method used or data acquired. The hit/miss POD model can be applied in all cases in which the presence or absence of detection can be classified. However, its application is limited in ordinary experimental conditions because a large number of samples are required to determine the reliability of the POD analysis result. On the other hand, the a^ vs. a POD model can usually be applied when the size of the acquired signal response data is proportional to the flaw size, and it is used to evaluate eddy current testing or UT [20]. Since signal response data have more subcategorized information than hit/miss data, they can produce effective POD analysis results, even with only a small number of samples [21,22]. In this study, therefore, we used the $\hat{a}\;$ vs. a POD model using signal response data to perform reliability analysis. For a^, we applied the measured flaw size (length and height) data. To apply the a^ vs. a POD model, the signal response data for the flaw size must satisfy the following four conditions: (a) the linearity of the parameters, (b) uniform variance, (c) uncorrelated observations, and (d) normal errors [21].

**Figure 5:**
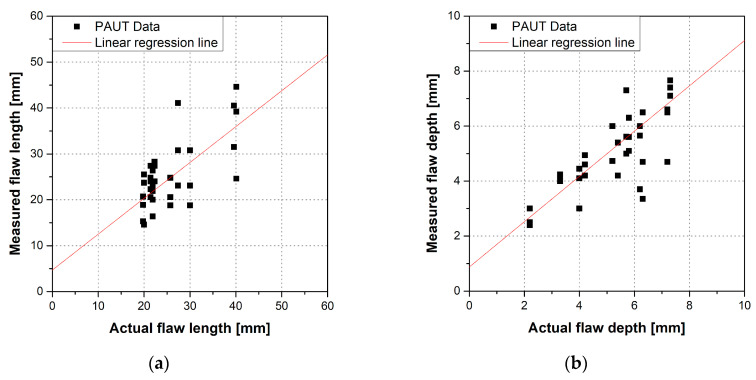
Linearity of actual defect size and PAUT measurements: (**a**) flaw length and (**b**) flaw depth.

We verified the validity of the measured flaw size data to apply the a^ vs. a POD model. Figure 5 shows the results of the linear regression analysis on the data acquired in the RRT. The correlation coefficient was calculated as 0.78 for flaw length and 0.82 for flaw height. Correlation coefficients above 0.7 are strong linear relationships. Thus, it can be concluded that the sizes of the actual and measured flaws exhibit a linear relationship. Furthermore, the variance of the PAUT data for the notches was calculated in a previous study, which confirmed that they have uniform variance and are uncorrelated observations [22].

To evaluate the normal distribution, quantile–quantile plots (QQ-plots) were calculated. A QQ-plot is a statistical analysis technique widely applied to evaluate the normality of two datasets. The difference between the actual defect size and the measured defect size is separated into the same probability interval and the percentile (z-score) of the standard normal distribution for each dataset is calculated. A QQ-plot is then obtained by plotting the difference and z-score of two datasets on the x-y plane.

**Figure 6:**
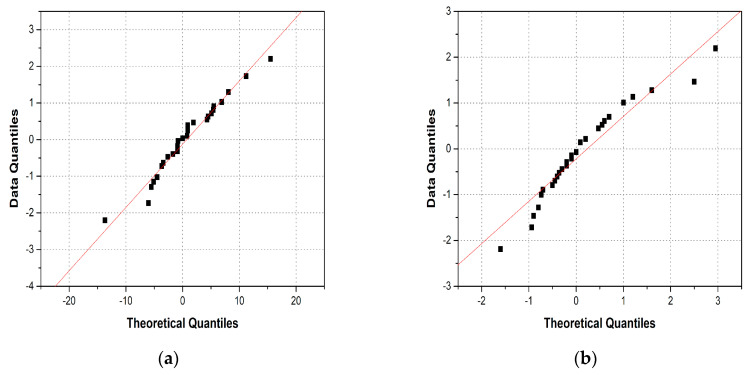
Quantile–quantile plots of the PAUT data: (**a**) flaw length and (**b**) flaw depth.

We calculated the QQ-plots in accordance with the RRT data, as shown in Figure 6. In a QQ-plot, the data distribution follows a normal distribution when it is close to a 45-degree line, where y = x [23]. Furthermore, the values of the coefficient of determination r are 0.97 and 0.95 for the flaw length and depth, respectively, showing a strong correlation. Thus, we determined that the data acquired in this study follow a normal distribution. Prior to the reliability analysis, we confirmed that the four conditions for applying the a^ vs. a POD model were satisfied.

**Figure 7:**
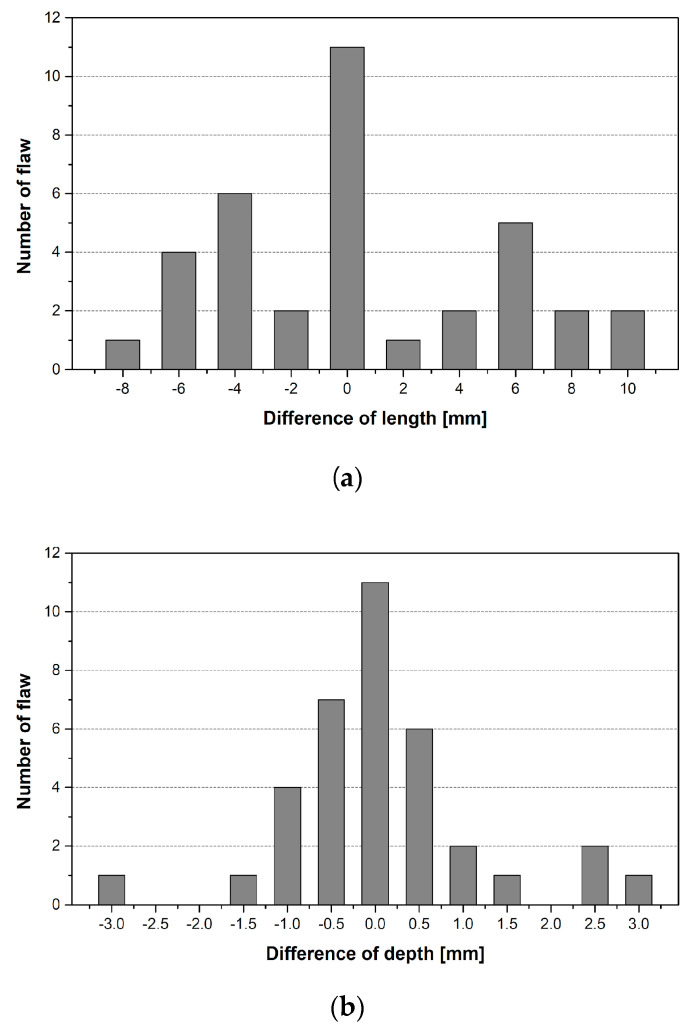
Differences between actual and measured flaw sizes in the thermal fatigue specimens: (**a**) flaw length and (**b**) flaw depth.

For the a^ vs. a POD analysis, we selected noise and decision threshold levels. These are determined based on a statistical method or the signal response data, and the result of the POD varies depending on these values [24]. In this study, we selected the decision threshold level for the signal response data by calculating the standard deviation of the difference between the actual and measured flaw sizes (a − a^) [25]. The noise level is a criterion to distinguish between the non-flaw and flaw signals; we applied the same value as the decision threshold level, which was a determination criterion. Figure 7 shows the variance of a − a^, which was used to determine the decision threshold level.

The standard distributions for the above variances are 5.63 mm and 1.69 mm for the flaw length and depth, respectively. We have explained the statistical basis for applying the $\hat{a}\;$ vs. a POD model and calculated the parameters required for the analysis. In the POD analysis, we calculated the POD for the flaw length and depth using the commercial program mh1823 [21] and compared the results to the acceptance criteria suggested in the ASME Code [7]. Figure 6 shows the $\hat{a}\;$ vs. a POD analysis result of the flaw lengths.

**Figure 8:**
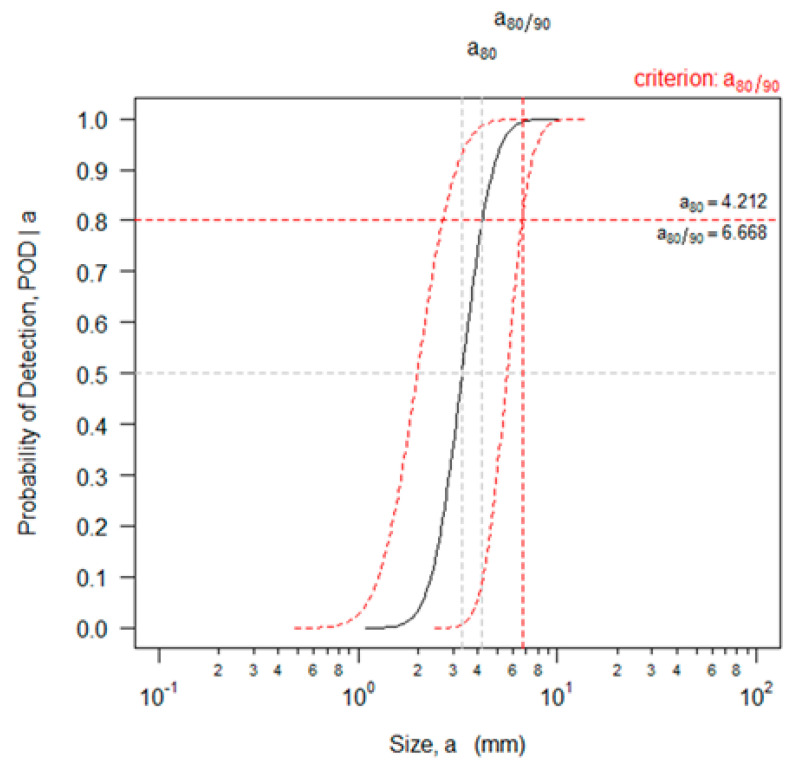
POD analysis result of flaw lengths for thermal fatigue specimens.

In the POD curve in Figure 8, the x-axis represents the flaw length, and the y-axis represents the POD. The black solid line represents the POD for the flaw size, and the red dotted line represents the confidence interval. In general, the POD converges to 0 as the flaw size decreases and approaches 1 as the flaw size increases. This analysis result shows a POD of 80% for a flaw of approximately 4.2 mm in length. The ASME Code case has defined the acceptable flaw size for UT in pipe welds with a thickness of 13 mm or greater. The flaws in the fabricated specimens are thermal fatigue cracks grown along the inner diameter, and if the acceptance criteria of surface flaws are applied, the acceptable flaw length is 0.25 in (6.4 mm). Therefore, the results of the POD analysis for length satisfied the ASME Code requirements. However, a80/90, i.e., the value of the 90% confidence interval, is approximately 6.6 mm, which exceeds the acceptable flaw length. In general, the UT method tends to show a large deviation in the results depending on the skill of the inspector. To confirm this, we calculated the 80% POD, and, in turn, a80 and a80/90 for each inspection team, which are listed in Table 2. All inspection teams used the same equipment and procedure to acquire data; however, the deviation was large. In the results of the POD analysis of Team 1, we found that the acceptable flaw length was satisfied, even at a confidence level of 90%. Teams 2 and 3 confirmed that the 80% POD satisfied the acceptance criteria, but the confidence interval was out of the acceptance criteria.

**Figure 9:**
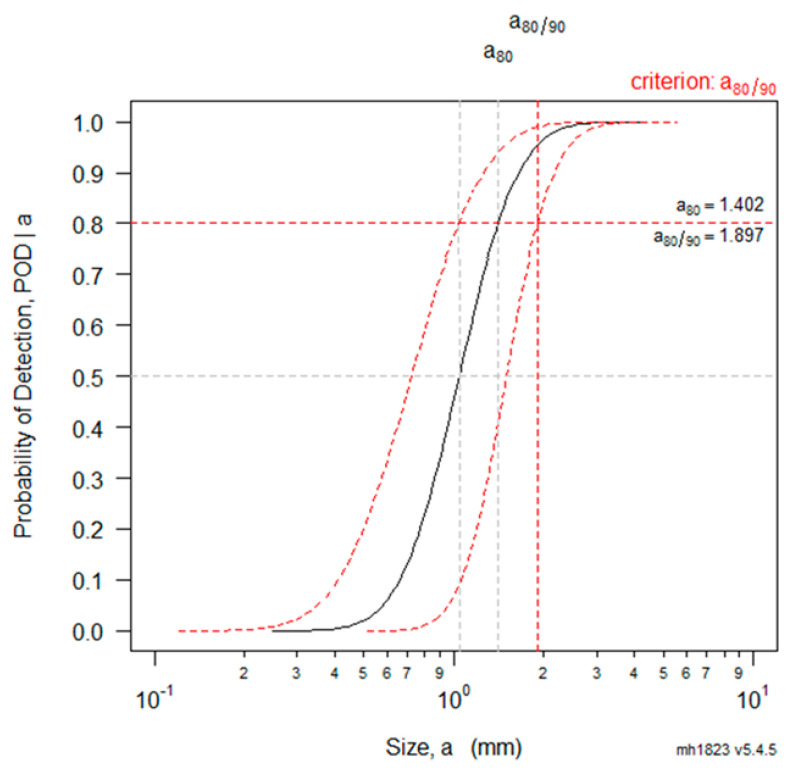
POD analysis result of flaw depths of thermal fatigue specimens.

Figure 9 shows the $\hat{a}\;$ vs. a POD analysis result of the flaw depth; the calculated a80 was approximated as 1.4 mm. The acceptable flaw height presented in the ASME Code is 0.087 or less when divided by the thickness of the inspection target. If a smaller specimen thickness is applied from a conservative perspective, a passing criterion of approximately 1.51 mm is calculated. As a result, we can conclude that the PAUT analysis result for the thermal fatigue crack pipes satisfies the acceptance criteria for both the length and depth. As with the analysis of the lengths, we calculated an 80% POD and a 90% confidence level of the flaw depths for each inspection team, which are summarized in Table 3.

Based on the 80% POD analysis result of each team, every inspection team satisfies the acceptance criteria. However, when considering the 90% confidence level, the deviation is large. Even if the 80% POD is at the same level, the confidence level should be calculated to check the reliability of the POD itself. This is related to the size measurement accuracy, which will now be described.

**Figure 10:**
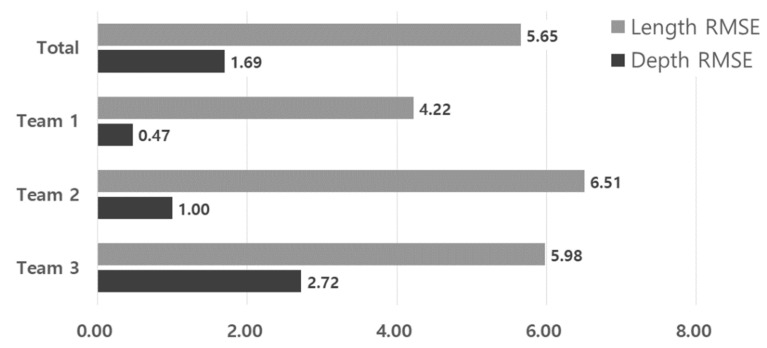
RMSE analysis results for flaw length and depth.

The root-mean-square error (RMSE) is a method of calculating the error between the reference data and the measurement data and is used to evaluate the size measurement accuracy in the nondestructive test. We referred to the ISI acceptance criteria of the ASME Code for the RMSE-based acceptance criteria, which are 19 mm for the flaw length and 3.175 mm for the depth; Figure 10 shows that all inspection teams satisfied the acceptance criteria. The POD and RMSE analysis results are summarized in Table 4.

## 4. Conclusions

In this study, the reliability of the PAUT method for thermal fatigue cracks was quantitatively evaluated by analyzing POD and sizing performance. The specimens were fabricated by growing cracks under thermal fatigue conditions as in the nuclear power plant environment, and PAUT data for thermal fatigue cracks were collected by performing an RRT with inspectors who were qualified for standardized equipment and procedures. The measured flaw length and height for thermal fatigue cracks were verified as having a statistically high correlation with the actual flaw size. It was confirmed that sizing data can be applied as a response parameter to the a^ vs. a POD model. By validating the a^ vs. a POD model, the accuracy of the reliability analysis can be improved by replacing the hit/miss POD model. As a result of the POD analysis, the detectable flaw length of 80% POD was 4.21 mm and the height was 1.41 mm, both of which satisfied the acceptable criteria of length, 6.4 mm, and height, 1.51 mm. In addition, as an evaluation result of sizing performance, the slope of the linear regression was 0.78 in length and 0.82 in height proving that there was a significant correlation. The RMSE satisfied the acceptance criteria (length 19 mm; height 3.175 mm) with 5.65 mm for flaw length and 1.69 mm for height. It was confirmed that the PAUT method can be applied to the inspection of thermal fatigue cracks of pipe welds in nuclear power plants. The reliability evaluation results of PAUT in this study represent the performance of a specific inspection system (inspector, equipment, or procedure). Depending on the application of other variables to the inspection system, the reliability evaluation result changes. Therefore, the PAUT technique for tube and pipe welds in power plant facilities could be used as technical evidence for applying PAUT to manufacturing or in-service inspection.

## Figures and Tables

**Table 1:** Experimental set-up for PAUT data acquisition.

Parameter	Value
**Probe Type**	1D-Array
**Wave Type**	Shear wave
**Law Configure**	Sectorial
**Focus Type**	True depth
**Aperture Area [** ${mm}^{2}$ **]**	Min. 8 × 10
**Sweep Angle [°]**	40~70
**Angle Resolution [°]**	1.0
**Focal Depth**	Bottom

**Table 2:** POD analysis result of flaw length by inspection team [mm].

Team No.	a_80_	a_80/90_
Total	4.21	6.66
Team 1	3.14	5.27
Team 2	5.05	10.48
Team 3	4.62	9.97

**Table 3:** POD analysis result of flaw depth by inspection team.

Team No.	a_80_	a_80/90_
Total	1.40	1.89
Team 1	1.38	1.60
Team 2	1.41	2.12
Team 3	1.36	3.64

**Table 4:** Comparison of RRT results against the ASME Code acceptance criteria.

Parameter	POD		RMSE	
	Acceptance Criteria	RRT Results	Acceptance Criteria	RRT Results
Length	6.4 mm	4.21 mm	19 mm	5.65 mm
Depth	1.51 mm	1.40 mm	3 mm	1.69 mm

## Data Availability

Not applicable.
